# Pharmacovigilance study on neurological adverse reactions of proteasome inhibitors in the FDA adverse event reporting system

**DOI:** 10.3389/fphar.2025.1712361

**Published:** 2026-01-05

**Authors:** Shuyue Li, Tao Ling, Yan Liu, Jun Li

**Affiliations:** 1 Department of Pharmacy, Shanxi Province Cancer Hospital/Shanxi Hospital Affiliated to Cancer Hospital, Chinese Academy of Medical Sciences/Cancer Hospital Affiliated to Shanxi Medical University, Taiyuan, China; 2 Department of Pharmacy, Suqian First Hospital, Suqian, China; 3 Department of Hematopathology, Shanxi Provincial Cancer Hospital/Shanxi Hospital Affiliated to Cancer Hospital, Chinese Academy of Medical Sciences/Cancer Hospital Affiliated to Shanxi Medical University, Taiyuan, China

**Keywords:** bortezomib, carfilzomib, ixazomib, FAERS, neurological adverse reaction

## Abstract

**Objective:**

Bortezomib, carfilzomib and ixazomib are the proteasome inhibitors (PIs) used to treat multiple myeloma (MM). We conducted a comprehensive pharmacovigilance analysis of their neurotoxicity using the Food and Drug Administration Adverse Event Reporting System (FAERS), including not only peripheral neurotoxicity but also central neurotoxicity, to provide reference for safe and rational clinical use.

**Methods:**

We obtained PIs’ adverse reaction reports during Q1 2004 to Q2 2025 from the FAERS database. Adverse drug event (ADE) signals of bortezomib, carfilzomib and ixazomib were analyzed by statistical methods including Reporting Odds Ratio (ROR), Proportional Reporting Ratios (PRR), Bayesian Confidence Propagation Neural Network (BCPNN) and Multi-item Gamma-Poisson Shrinker (MGPS). ADEs sorted by frequency of occurrence and signal strength. Subgroup analyses based on gender was performed to explore differences. Time-to-onset profiles were analyzed using the Weibull Shape Parameter (WSP) test.

**Results:**

A total of 33,322, 14,063, and 16,562 ADEs of bortezomib, carfilzomib and ixazomib were analyzed, respectively from the FAERS database. The most common neurological adverse reaction signals for bortezomib, carfilzomib, and ixazomib are peripheral neuropathy (PN), with bortezomib having the highest number of reports (n = 2,681). Analysis shows that compared to the other two drugs, bortezomib exhibits higher signal intensity in neurological adverse events. The most prominent signal of bortezomib is autonomic neuropathy [n = 96; ROR 70.16 (95% CI 56.79–86.67)]. The strongest signal of carfilzomib is in hypertensive cephalopathy [n = 7; ROR 18.09 (95% CI 8.58–38.12)], while ixazomib has the highest signal in burning feet syndrome [n = 3; ROR 16.61 (95% CI 5.32–51.91)]. The median to onset time for neurological adverse events related to bortezomib, carfilzomib, and ixazomib were 33days (IQR 13–91), 35 days (IQR 9–145), and 80 days (IQR 19–251), respectively.

**Conclusion:**

In real-world pharmacovigilance studies, the peripheral neurotoxicity of carfilzomib and ixazomib was lower than that of bortezomib. In addition to PN, it is necessary to pay more attention to the special central neurological adverse events of PIs, such as posterior reversible encephalopathy syndrome (PRES) related to bortezomib and carfilzomib, and hypertensive encephalopathy related to carfilzomib, for early symptom identification and diagnosis. For ixazomib, attention should also be paid to neuromuscular symptoms and prevention of neuralgia caused by herpes zoster virus.

## 1 Introduction

Proteasome inhibitors (PIs) are a class of revolutionary anti-tumor drugs. PIs induce tumor cell apoptosis, inhibit angiogenesis, and kill tumor cells by targeting and inhibiting the activity of proteasomes, interfering with the degradation process of intracellular proteins (Gazzaroli et al., 2023). Since bortezomib was approved in 2003, PIs have become the core drug for the treatment of multiple myeloma (MM), significantly improving the response rate and survival of patients. At present, the commonly used PIs in clinical practice include the first-generation bortezomib, the second-generation carfilzomib, and the oral formulation ixazomib.

While PIs have demonstrated remarkable efficacy in MM treatment, their associated adverse effects continue to pose significant clinical challenges. As is well known, bortezomib is frequently associated with peripheral neuropathy (PN) (Yang et al., 2024). Severe PN may present with diverse symptoms, including sensory disturbances, burning sensations, dysesthesia, neuropathic pain, and fatigue, all of which can substantially impair patients’ daily functioning, diminish quality of life, and compromise treatment adherence. Clinical trial data indicate that the overall incidence of bortezomib-induced PN ranged from 8.4% to 80.5% (median 37.8%) and severe neuropathy (grade 3–4) ranged from 1% to 33.2% (median 8%) (Li et al., 2020). Compared to this, carfilzomib and ixazomib are believed to exhibit reduced neurotoxicity (Kaplan et al., 2017; Schlafer et al., 2017).

It is worth noting that there is currently a lack of head-to-head comparative studies on the neurological safety of different PIs drugs, and there is no comprehensive systematic analysis based on large databases. This study systematically analyzed the neurological adverse drug events (ADEs) of bortezomib, carfilzomib, and ixazomib extracted from the FDA Adverse Event Reporting System (FAERS) database, including not only peripheral neurotoxicity but also central nervous system toxicity, and delved into the characteristics, signal intensity, and potential differences of neurotoxicity of PIs in real-world populations. The research aims to provide evidence-based reference for optimizing clinical medication strategies and strengthening drug safety monitoring, it has important clinical significance and public health value.

## 2 Materials and methods

### 2.1 Data sources

The data for this study were sourced from ADE reports in the FAERS database, covering Q1 2004 to Q2 2025. FAERS is an open-access database that tracks the safety profiles of drugs on the market. It compiles ADE reports from various sources, including healthcare professionals, pharmaceutical manufacturers, consumers, and so on. Each entry in the database details the patient’s demographics, suspected drugs, reported ADEs, patient outcomes, and the timing of ADE occurrences. The FAERS data used in this study were collected and preprocessed by R software (version 4.2.3), involved the collection and cleaning of the data. This process included encoding and classifying the preferred terms (PTs) and system organ class (SOC), which were recorded in the Medical Dictionary for Regulatory Activities (MedDRA).

### 2.2 Data extraction and identification

Bortezomib, carfilzomib and ixazomib were searched for their generic names and drug product names, respectively, including VELCADE, KYPROLIS and NINLARO. We screened all ADE reports in the FAERS database that identified PIs as the primary suspected drug, collected their PT and SOC, regardless of the reported indications, to fully capture real-world safety. The sex, age, and reporting country were also included in the analysis. According to the recommendation, we have implemented a duplicate data elimination procedure to ensure that each adverse event case is only counted once. The flowchart of adverse event screening is shown in Supplementary Figure S1.

### 2.3 Statistical analysis

In this study, the descriptive statistics were used to present ADE reports related to bortezomib, carfilzomib and ixazomib, and the disproportionality analysis was used to explore the potential association between these PIs and ADEs, a standard approach in pharmacovigilance research, including ①Reporting Odds Ratio (ROR), ②Proportional Reporting Ratios (PRR), ③Bayesian confidence propagation neural network (BCPNN) and ④Multi-item Gamma-Poisson Shrinker (MGPS) (van Puijenbroek et al., 2002). Each method has distinct strengths. ROR excels in mitigating biases from small samples and is highly effective for early signal detection. PRR performs reliably with incomplete datasets. BCPNN boosts signal detection accuracy by integrating multi-source data and cross-validation, remaining powerful even with few reports. MGPS specializes in identifying rare event signals while minimizing random patterns. Combining these four complementary methods ensures more reliable and robust results, effectively offsetting the limitations of individual approaches. The fourfold table and equations are described in Supplementary Tables S1, S2. The data that were chosen for analysis in our study were ADE signals that met four algorithm standards. Higher ROR values in the calculations denote a stronger signal intensity, which implies a stronger statistical association between the drug and the ADE. We sorted the occurrence frequency and signal strength of ADEs separately and identified ADEs that may have clinical significance. It must be pointed out that the FAERS database is a spontaneous reporting system with inherent limitations, including reporting bias, incomplete data, etc. The report cannot determine the causal relationship between the medication and adverse events. The disproportionate analysis used in this article aims to screen for strong statistical association signals, rather than confirming causal relationships.

Additionally, to explore ADE signals of populations with different characteristics, we used the ROR method for subgroup analysis based on gender (male, female) after excluding data with incomplete information. Based on a 2 × 2 contingency table, calculate the ROR value and use chi square (χ^2^) test to calculate the p-value. Create a volcano chart, with the X-axis displaying the ROR value converted from log2 and the Y-axis displaying the p-value converted from - log10. All data processing and statistical analyses were performed using R software 4.2.3.

### 2.4 Cumulative incidence and time-to-onset

The time onset analysis is defined as the time from the start of PIs treatment to the occurrence of neurological adverse events. Select reports with data time-to-onset, use the Kaplan Meier method to plot the cumulative incidence of adverse events, and use the log-rank test. Use the Weibull Shape Parameter (WSP) test for analysis of onset time. The WSP test can determine the rate of change in the incidence of adverse events (Sauzet et al., 2013). The shape parameter β of Weibull distribution determines the shape of the distribution function (Kinoshita et al., 2020): when the shape parameter β < 1 and its 95% CI < 1, it is considered that the risk decreases over time (early failure type profile); When the shape parameter β is equal to or close to 1 and its 95% CI includes a value of 1, it is estimated that the hazard continues to occur over time (random failure type curve); When the shape parameter β > 1 and its 95% CI does not include the value 1, it is considered that the danger increases over time (wear failure type curve).

### 2.5 Sensitivity analysis

Combining literature and searching the FAERS database, it was found that in the process of using PIs, other drugs that may cause neurotoxicity may be used in combination. Based on the frequency of concomitant drug occurrence, we excluded reports of concomitant use of thalidomide. Sensitivity analysis was conducted to confirm the robustness of the data.

## 3 Results

### 3.1 Descriptive results for the total population

**FIGURE 1:**
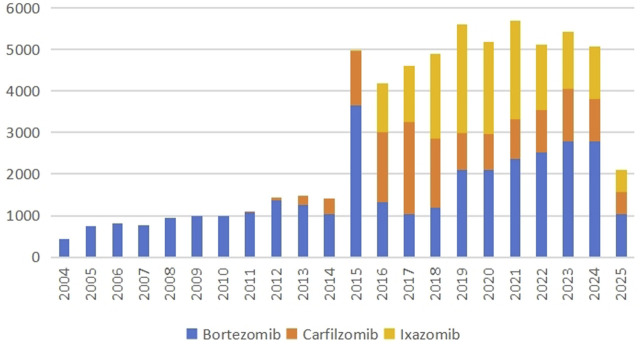
Annual adverse reaction reports of bortezomib, carfilzomib and ixazomib from Q1 2004 to Q2 2025.

ADE reports from Q1 2004 to Q2 2025 were collected from the FAERS database. There were 33,322 reports of bortezomib as the main suspected drug, 14,063 reports of carfilzomib as the main suspected drug, and 16,562 reports of ixazomib, as shown in Table 1. The number of adverse reaction reports per year is shown in Figure 1. Bortezomib has been on the market for the longest time, therefore, it has the highest number of ADE reports. Although ixazomib was launched later than carfilzomib, the number of ADEs was higher than that of carfilzomib. Except for data that cannot be obtained, the gender ratio is comparable. In terms of age, since MM is more common in elderly people, ADEs of three types of PI preparations are most concentrated in the 65–85 age group. From the perspective of the reporting source, the reports are mainly from physicians, followed by those from consumers, health professionals, and pharmacists. ADE reports are concentrated in the United States, followed by countries such as Japan and France. Adverse drug reactions of PIs can lead to prolonged hospitalization in about 20% of patients, with the highest percentage in ixazomib (27.1%). But fewer adverse reactions lead to disability.

**TABLE 1:** Baseline characterization of three proteasome inhibitors.

Characteristics	Bortezomib	Carfilzomib	Ixazomib	Total
	n = 33,322	n = 14,063	n = 16,562	n = 63,947
Gender				
Female	10,386 (31.2%)	4,768 (33.9%)	7,514 (45.4%)	22,668 (35.4%)
Male	13,409 (40.2%)	6,010 (42.7%)	8,256 (49.8%)	27,675 (43.3%)
Missing	9,527 (28.6%)	3,285 (23.4%)	792 (4.8%)	13,604 (21.3%)
Age (years)				
<18	993 (3.0%)	138 (1.0%)	414 (2.5%)	1,545 (2.4%)
18–64.9	8,229 (24.7%)	4,055 (28.8%)	2,538 (15.3%)	14,822 (23.2%)
65–85	9,575 (28.7%)	4,937 (35.1%)	5,978 (36.1%)	20,490 (32.0%)
>85	382 (1.1%)	127 (0.9%)	545 (3.3%)	1,054 (1.6%)
Missing	14,143 (42.4%)	4,806 (34.2%)	7,087 (42.8%)	26,036 (40.7%)
Report source				
Consumer	5,425 (16.3%)	1,380 (9.8%)	5,431 (32.8%)	12,236 (19.1%)
Health professional	3,176 (9.5%)	2,105 (15.0%)	1,359 (8.2%)	6,640 (10.4%)
Pharmacist	1,771 (5.3%)	1,089 (7.7%)	1,257 (7.6%)	4,117 (6.4%)
Physician	18,240 (54.7%)	7,391 (52.6%)	6,347 (38.3%)	31,978 (50.0%)
Missing	5,425 (16.3%)	1,380 (9.8%)	5,431 (32.8%)	8,976 (14.0%)
Report country				
United States	11,858 (35.6%)	8,306 (59.1%)	8,929 (53.9%)	29,093 (45.5%)
France	2,426 (7.3%)	686 (4.9%)	592 (3.6%)	3,704 (5.8%)
Japan	2,016 (6.0%)	678 (4.8%)	1,514 (9.1%)	4,208 (6.6%)
Australia	2,204 (6.6%)	296 (2.1%)	55 (0.3%)	2,555 (4.0%)
Germany	1,638 (4.9%)	530 (3.8%)	61 (0.4%)	2,229 (3.5%)
China	1,214 (3.6%)	258 (1.8%)	1,146 (6.9%)	2,618 (4.1%)
Outcome				
Death	4,137 (12.4%)	2,466 (17.5%)	3,627 (21.9%)	10,230 (16.0%)
Disability	173 (0.5%)	87 (0.6%)	37 (0.2%)	297 (0.5%)
Hospitalization	5,076 (15.2%)	3,360 (23.9%)	4,492 (27.1%)	12,928 (20.2%)
Life-threatening	694 (2.1%)	684 (4.9%)	180 (1.1%)	1,558 (2.5%)
Other	23,241 (69.7%)	7,466 (53.1%)	8,226 (49.7%)	38,933 (60.9%)

### 3.2 High frequency neurological PTs of PIs

After categorizing the adverse reaction reports of the three drugs into PTs and SOCs, we selected records classified under “nervous system disorders.” Bortezomib has a total of 9,013 reported cases of neurological adverse events, carfilzomib has 2,119 reported cases, and ixazomib has 3,450 reported cases. As shown in Table 2, the top ten most frequently reported nervous system PTs are listed. The signal strength calculated by the four statistical methods is shown in Supplementary Table S3. Peripheral neuropathy, the most prominent adverse reaction associated with PIs, exhibited the highest incidence among the three drugs. Bortezomib had the greatest number of reported cases (n = 2681, 29.7%), while ixazomib (n = 675, 19.6%) reported more occurrences than carfilzomib (n = 248, 11.7%). Among the top ten adverse reactions, autonomic neuropathy—a subtype of peripheral neuropathy—was reported for all three drugs. Bortezomib had the highest incidence (n = 96, 1.1%), while carfilzomib (n = 4, 0.2%) and ixazomib (n = 5, 0.1%) showed significantly fewer cases. Additionally, both bortezomib and carfilzomib have two high-frequency PTs: “polyneuropathy” and “posterior reversible encephalopathy syndrome”. While “peripheral sensory neuropathy” appeared simultaneously in the top 10 PTs of bortezomib and ixazomib.

**TABLE 2:** Top 10 neurological PTs with the highest frequency of occurrence.

Bortezomib (n = 9,013)		Carfilzomib (n = 2,119)		Ixazomib (n = 3,450)	
PT	n (%)	PT	n (%)	PT	n (%)
Neuropathy peripheral***	2,681 (29.7%)	Neuropathy peripheral***	248 (11.7%)	Neuropathy peripheral***	675 (19.6%)
Polyneuropathy**	393 (4.4%)	Polyneuropathy**	58 (2.7%)	Paraesthesia	252 (7.3%)
Neurotoxicity	221 (2.4%)	Posterior reversible encephalopathy syndrome**	53 (2.5%)	Dementia	74 (2.1%)
Peripheral sensory neuropathy**	171 (1.9%)	Encephalopathy	43 (2.0%)	Neurological symptom	31 (0.9%)
Neuralgia	117 (1.3%)	Hypertensive encephalopathy	7 (0.3%)	Ataxia	31 (0.9%)
Autonomic neuropathy***	96 (1.1%)	Intracranial mass	5 (0.2%)	Myoclonus	30 (0.9%)
Posterior reversible encephalopathy syndrome**	87 (1.0%)	Autonomic neuropathy***	4 (0.2%)	Peripheral sensory neuropathy**	17 (0.5%)
Guillain-barre syndrome	72 (0.8%)	Cauda equina syndrome	4 (0.2%)	Chorea	16 (0.5%)
Peripheral motor neuropathy	53 (0.6%)	Central nervous system haemorrhage	3 (0.1%)	Post herpetic neuralgia	6 (0.2%)
Peripheral sensorimotor neuropathy	45 (0.5%)	Cerebral small vessel ischaemic disease	3 (0.1%)	Autonomic neuropathy***	5 (0.1%)

***: indicates that this PT, appears three times in the table, **: indicates that this PT, appears twice in the table.

### 3.3 The neurological PT with high signal strength of PIs

**FIGURE 2:**
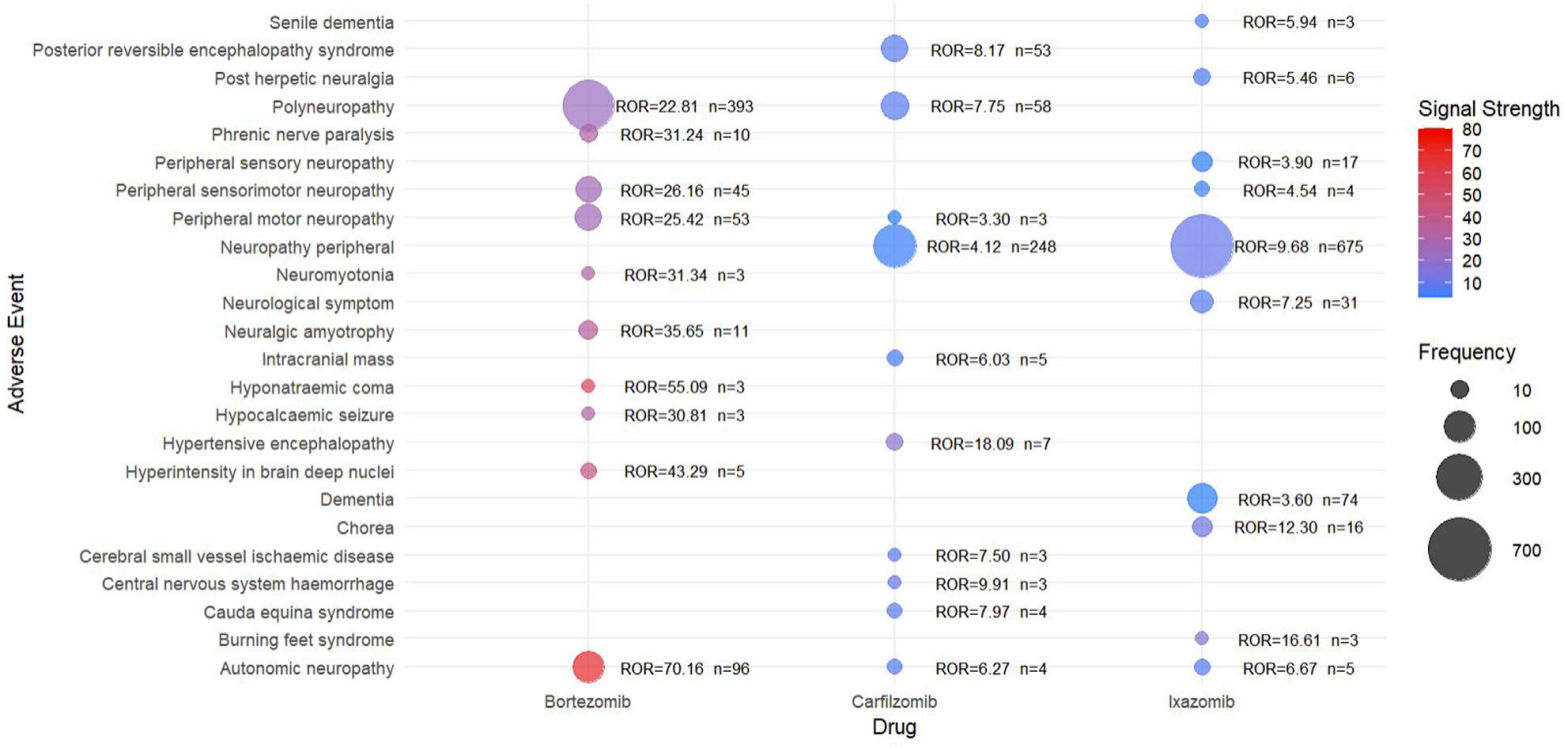
The top 10 neurological adverse events with the strongest ROR signal intensity (The color gradient of the bubbles represents the signal intensity, denoted by ROR, with the ROR value on the right side of the bubbles. The size of bubbles indicates the frequency of adverse events.).

We identified the top 10 PTs with the strongest safety signals among neurological adverse events associated with bortezomib, carfilzomib, and ixazomib (Supplementary Table S4). Using the ROR as a measure, we generated a bubble plot to visualize the signal intensities (Figure 2). Comparing the colors of bubbles, it can be observed that bortezomib demonstrated higher signal intensities compared to the other two proteasome inhibitors. The most prominent signal for bortezomib was autonomic neuropathy [n = 96; ROR 70.16 (95% CI 56.79–86.67)]. For carfilzomib, the strongest signal was hypertensive cephalopathy [n = 7; ROR 18.09 (95% CI 8.58–38.12)], while ixazomib showed its highest signal for burning feet syndrome [n = 3; ROR 16.61 (95% CI 5.32–51.91)].

### 3.4 Subgroup analysis

**FIGURE 3:**
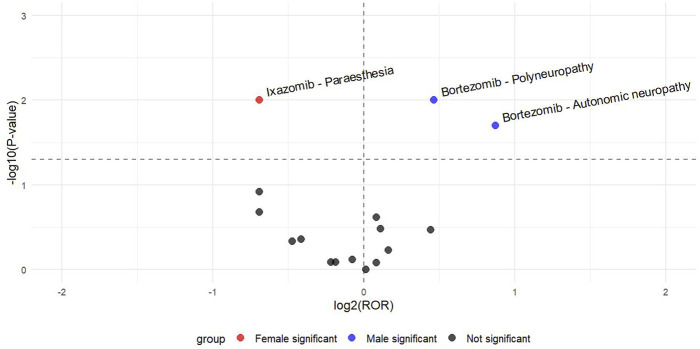
Sex difference risk signal volcano map.

To investigate gender differences in adverse event signals, a volcano map was created as a visual representation. In Figure 3, “Polyneuropathy” and “Autonomic Neuropathy” in bortezomib are associated with a higher risk of occurrence in males, while female patients taking ixazomib are more likely to experience paraesthesia. There is no gender difference in the other high-frequency ADE signals.

### 3.5 Sensitivity analysis

After excluding the reports of combined use of thalidomide, the frequency and signal strength of PT reports remained consistent with the previous analysis. See Supplementary Tables S3, S4. This proves the robustness of the data research in this article and the reliability of the conclusions.

### 3.6 Time to onset analysis

**FIGURE 4:**
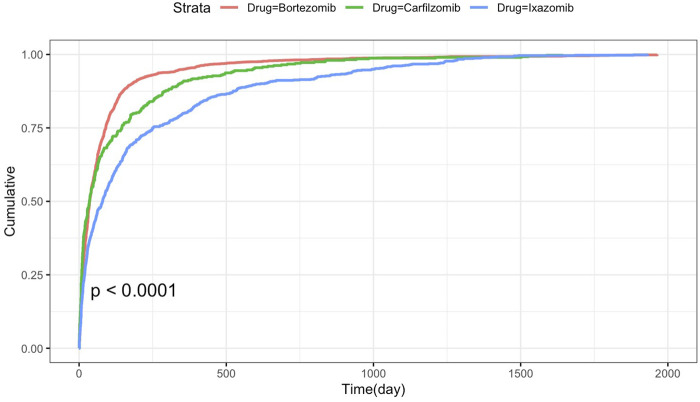
Time to onset of neurological adverse events under proteasome inhibitor treatment.

The summary of WSP test results for the three drugs is shown in Table 3. The median to onset time for neurological adverse events related to bortezomib, carfilzomib, and ixazomib were 33 days (IQR range: 13–91), 35 days (IQR range: 9–145), and 80days (IRQ range: 19–251), respectively. In the WSP analysis of PI-associated neurological adverse events, the upper limit of the 95% confidence interval for the shape parameter was below 1 (β < 1), indicating a decreasing hazard profile characteristic of early failure types. This suggests that the incidence of these adverse events tends to decline over time. The Kaplan Meier curve of PIs induced neurological adverse events is shown in Figure 4. There is a significant difference in the time to onset of neurological adverse events among bortezomib, carfilzomib, and ixazomib (log rank test, P < 0. 0001).

**TABLE 3:** Weibull shape parameter test for neurological adverse events of PI drugs.

Drug	Case	TTO (days)		Scale parameter		Shape parameter		Type
	n	Median (IQR)	Min-max	α	95%CI	β	95%CI	
Bortezomib	2,053	33 (13–91)	1–1,964	68.8	64.3–73.2	0.70	0.68–0.73	Early failure
Carfilzomib	399	35 (9–145)	1–1,644	81.9	67.8–95.9	0.61	0.56–0.65	Early failure
Ixazomib	794	80 (19–251)	1–1,932	153.5	136.1–171.0	0.65	0.61–0.68	Early failure

TTO: time to onset, IQR: interquartile range.

## 4 Discussion

The neurotoxicity of chemotherapy drugs refers to the fact that chemotherapy drugs affect the central nervous system and peripheral nervous system to produce corresponding symptoms (Was et al., 2022). This study represents first pharmacovigilance investigations utilizing real-world data from the FAERS database to characterize neurological adverse events associated with PIs. Using comprehensive FAERS data, we identified neurological ADEs significantly associated with bortezomib, carfilzomib, and ixazomib therapies. Furthermore, we analyzed the clinical characteristics of these adverse event reports to provide evidence-based guidance for optimizing PIs medication safety in clinical practice.

The utility of bortezomib as first-line therapy for MM–particularly in long-term treatment regimens–is limited by PN, which frequently manifests as severe neuropathic pain. According to reports, the mechanism of dose-dependent PN may be related to microtubule dysfunction and axonal injury (Velasco et al., 2019). Although bortezomib does not cross the blood-brain barrier (BBB) to directly access the central nervous system (CNS), it accumulates in dorsal root ganglia (DRG). This accumulation triggers glial cell activation, disrupts glutamate homeostasis, and induces neuroinflammation, ultimately resulting in indirect CNS dysfunction and neurotoxicity (Yamamoto and Egashira, 2021). Several pathological aspects have been observed, such as cytoskeletal damage, morphological changes of mitochondria and endoplasmic reticulum (ER), oxidative stress, sensitization of transient receptor potential ankyrin 1 (TRPA1), and neuroinflammation (Malacrida et al., 2021). The incidence and severity of PN depend on the cumulative drug dose, dosing regimen, and route of administration.

According to the data mining of the FAERS database, the main neurological adverse reactions of bortezomib in the real world are mainly peripheral nervous system lesions. include neuropathy peripheral (n = 2,681, ROR = 19.68, 95% CI 18.93–20.47), polyneuropathy (n = 393, ROR = 22.81, 95% CI 20.62–25.23), peripheral sensory neuropathy (n = 171, ROR = 19.95, 95% CI 17.13–23.24) and neuralgia (n = 117, ROR = 3.18, 95% CI 2.65–3.81). In subgroup analysis, male patients using bortezomib appear to have a higher risk of experiencing such adverse reactions. But most patients have mild peripheral nerve symptoms that do not require treatment (Messinger et al., 2010). At present, the prevention measures for PN include reducing the dose and using subcutaneous rather than intravenous administration (Moreau et al., 2011). The treatment drugs for PN mainly include B vitamins, levocarnitine, ganglioside, etc., but these treatment methods are mostly lacking large samples and randomized trials.

Notably, bortezomib was associated with clinically significant incidences of Posterior Reversible Encephalopathy Syndrome (PRES; n = 87, ROR = 5.65, 95% CI 4.57–6.97) and Guillain-Barre syndrome (GBS; n = 72, ROR = 10.15, 95% CI 8.04–12.82). PRES is a neuroradiological syndrome characterized by vasogenic edema involving the posterior occipital cortex and subcortical white matter, resulting in visual impairment, seizures, and altered mental status. Nixon and Parhar (2014) documented a case of bortezomib-induced PRES and conducted a comparative analysis with four previously reported cases. All five cases developed PRES within 1–2 treatment cycles, presenting with characteristic symptoms including altered consciousness and seizure activity. While PRES is generally reversible upon timely discontinuation of the causative agent and typically resolves without permanent neurological sequelae, failure to promptly recognize and manage this condition may result in severe complications, including intracranial hemorrhage or ischemic infarction, with potentially life-threatening consequences (Liu et al., 2021). Medical personnel should remain highly vigilant for early symptoms of PRES, such as sudden headaches, epilepsy, blurred consciousness, and visual impairments. Once relevant symptoms appear, diagnostic evaluation should be initiated immediately.

GBS is an immune-mediated acute inflammatory peripheral neuropathy. It is characterized by the involvement of multiple nerve roots and peripheral nerve injury, manifested by rapidly progressive muscle weakness, paresthesia and areflexia. In severe cases, it can involve respiratory muscles, resulting in dyspnea and even life-threatening. In this study, only bortezomib had GBS-related adverse events among PI drugs, and the mechanism is still unclear. There have been previous reports of GBS after bortezomib treatment. One MM patient developed sensory abnormalities in the upper and lower limbs 5 days after bortezomib and dexamethasone treatment, and the diagnosis of GBS was considered (Dai et al., 2015). Subsequently, the patient received high-dose intravenous immunoglobulins (IVIGs 400 mg/kg/day for 5 days), and the symptoms were basically relieved. Another case report was similar. It occurred 14 days after the second course of treatment (Xu et al., 2019). After receiving high-dose IVIG treatment, the symptoms were completely relieved without recurrence. Li et al. (2025) reported that 3 MM patients were diagnosed with GBS during bortezomib treatment. Two of the three patients had a clear history of upper respiratory tract infection before the onset of GBS. After intravenous immunoglobulin treatment, one patient died, and GBS symptoms improved in two patients. The relationship between the etiology of MM complicated with GBS and bortezomib is unclear, but clinicians need to be vigilant. For patients using bortezomib, early differential diagnosis of GBS is necessary when they suddenly experience unexplained limb weakness, especially when combined with infection.

Compared to bortezomib, carfilzomib demonstrated a significantly lower incidence of PN, as evidenced by FAERS database reports. The reported cases and corresponding signal strengths were substantially reduced: neuropathy peripheral (n = 248; ROR = 4.12, 95% CI 3.64–4.67), polyneuropathy (n = 58; ROR = 7.75, 95% CI5.99–10.04), and autonomic neuropathy (n = 4; ROR = 6.27, 95% CI 2.35–16.73). However, carfilzomib was associated with central nervous system manifestations including headache and dizziness. Notably, 53 cases of PRES were documented in the FAERS database (ROR = 8.17, 95% CI 6.24–10.70). There are also relevant case reports in the literature (Kadhem et al., 2017), suggesting the possibility of patients receiving treatment with carfilzomib developing status epilepticus and the necessity of timely differential diagnosis of PRES.

The risk of hypertension caused by carfilzomib is the highest among the three drugs. Clinical studies indicate that after four treatment cycles, carfilzomib induces significant blood pressure elevation, with mean increases of 10 mmHg in systolic blood pressure, 3.3 mmHg in diastolic blood pressure, and 5.4 mmHg in mean arterial pressure, potentially exacerbating pre-existing hypertension (Gavazzoni et al., 2018). The risk of hypertension caused by bortezomib and ixazomib is low (Wu et al., 2020). Our analysis of FAERS data identified hypertensive encephalopathy as the adverse event with the strongest safety signal for carfilzomib (n = 7; ROR = 18.09, 95% CI 8.58–38.12), underscoring the critical need for rigorous blood pressure monitoring during carfilzomib therapy.

Besides, prescribing information of carfilzomib alerts clinicians to serious cerebrovascular risks including intracranial hemorrhage and stroke. Real-world data reports 3 cases of central nervous system haemorrhage (ROR 9.91; 95% CI 3.19–30.86) and 3 cases of cerebral small vessel ischaemic disease (ROR 7.50; 95% CI 2.41–23.31). While these events remain uncommon, their moderate signal strength and potentially life-threatening nature demand careful clinical monitoring during treatment.

As an oral PI, ixazomib also demonstrates a lower incidence of PN compared to bortezomib based on FAERS data analysis. The reported cases include neuropathy peripheral (n = 675; ROR 9.68, 95% CI 8.97–10.45) and paresthesia (n = 252; ROR 2.10, 95% CI 1.85–2.37). “Paresthesia” exhibits a gender bias, being more prevalent in female patients, which is a noteworthy clinical feature. However, ixazomib may induce neuromuscular manifestations such as muscle weakness and coordination disorders in some patients, potentially through effects on motor nerve function in the peripheral nervous system. Studies have shown that bortezomib can lead to reversible muscle weakness, and the mechanism may be the accumulation of lipid droplets in muscle fibers, as well as mitochondrial alterations consisting of swelling and cristae loss at ultrastructural examination (Pancheri et al., 2020). Our analysis of the FAERS database identified 31 cases of ataxia (ROR 3.40, 95% CI 2.39–4.83) and 30 cases of myoclonus (ROR 3.30, 95% CI 2.31–4.72). These findings, demonstrating moderate signal strengths, suggest ixazomib may have a propensity for inducing motor nerve dysfunction.

The FAERS database analysis identified 73 cases of dementia associated with ixazomib (ROR 3.60, 95% CI 2.87–4.53). Although this adverse reaction is not recorded in the instructions, it has also been found in previous studies that cognitive disorder manifests as a high signal of ixazomib (Luo et al., 2025). At present, research has only explored the mechanism of cognitive dysfunction caused by bortezomib induced damage to cerebral vascular endothelial cells (Lu et al., 2023), and it is unclear whether ixazomib is similar to it. Additionally, post-herpetic neuralgia was reported in 6 cases (ROR 5.46, 95% CI 2.45–12.18), demonstrating notable signal strength. Both the prescribing information and clinical studies recommend prophylactic antiviral therapy for patients receiving ixazomib to mitigate the risk of herpes zoster virus reactivation.

To examine the onset characteristics of PIs’ related neurological adverse events, time to onset analysis was performed, and the results showed significant differences among PIs (P < 0.0001). Bortezomib demonstrated the shortest median time to symptom onset (33 days), followed by carfilzomib (35 days), with ixazomib showing the longest latency period (80 days). WSP test showed the neurotoxicity of the three PIs all showed an early failure model, indicating that the risk of PIs related neurologic adverse events decreased over time. On the one hand, it means that PIs related neurologic adverse events mostly occur in the early stage of treatment. On the other hand, considering that PIs related to PN are a dose cumulative adverse reaction, this curve may mean the reduction or withdrawal of PI drugs due to adverse reactions.

Our study had several limitations. Due to the spontaneous reporting of information in the FAERS database, and the fact that not all personnel involved in reporting are medical staff, the quality of reporting is also uneven. Therefore, there are often deficiencies such as missing or omitted reporting data, a lack of actual clinical information of patients, and difficulty in ruling out other risk factors. In addition, the positive signals identified in this study can only indicate a statistical correlation between the drug and ADE signals, but their clear clinical significance still needs to be verified by real-world studies. Furthermore, the geographical distribution of FAERS reports is skewed, with the majority originating from western countries. Therefore, the generalizability of our findings to patient populations in other regions, such as Asia or Africa, may be limited and requires further investigation.

Overall, comparative pharmacovigilance analysis showed that compared with the first generation bortezomib, the second generation (carfilzomib) and the third generation (ixazomib) PIs showed delayed onset time and significantly reduced neurotoxicity, especially in the incidence of PN. In clinical management, it is necessary to pay more attention to the special adverse events of PIs, such as bortezomib related PRES, GBS, carfilzomib related hypertensive encephalopathy and PRES, as well as neuromuscular symptoms and herpes zoster related neuralgia caused by ixazomib.

## Data Availability

The raw data supporting the conclusions of this article will be made available by the authors, without undue reservation.
