# Supplementary material for: Pharmacovigilance study on neurological adverse reactions of proteasome inhibitors in the FDA adverse event reporting system
Source: Front Pharmacol. 2026 Jan 5;16:1712361. doi: 10.3389/fphar.2025.1712361 (PMC12812994; doi:10.3389/fphar.2025.1712361)
Supplement: Supplementary file 1 [file Supplementaryfile1.docx]

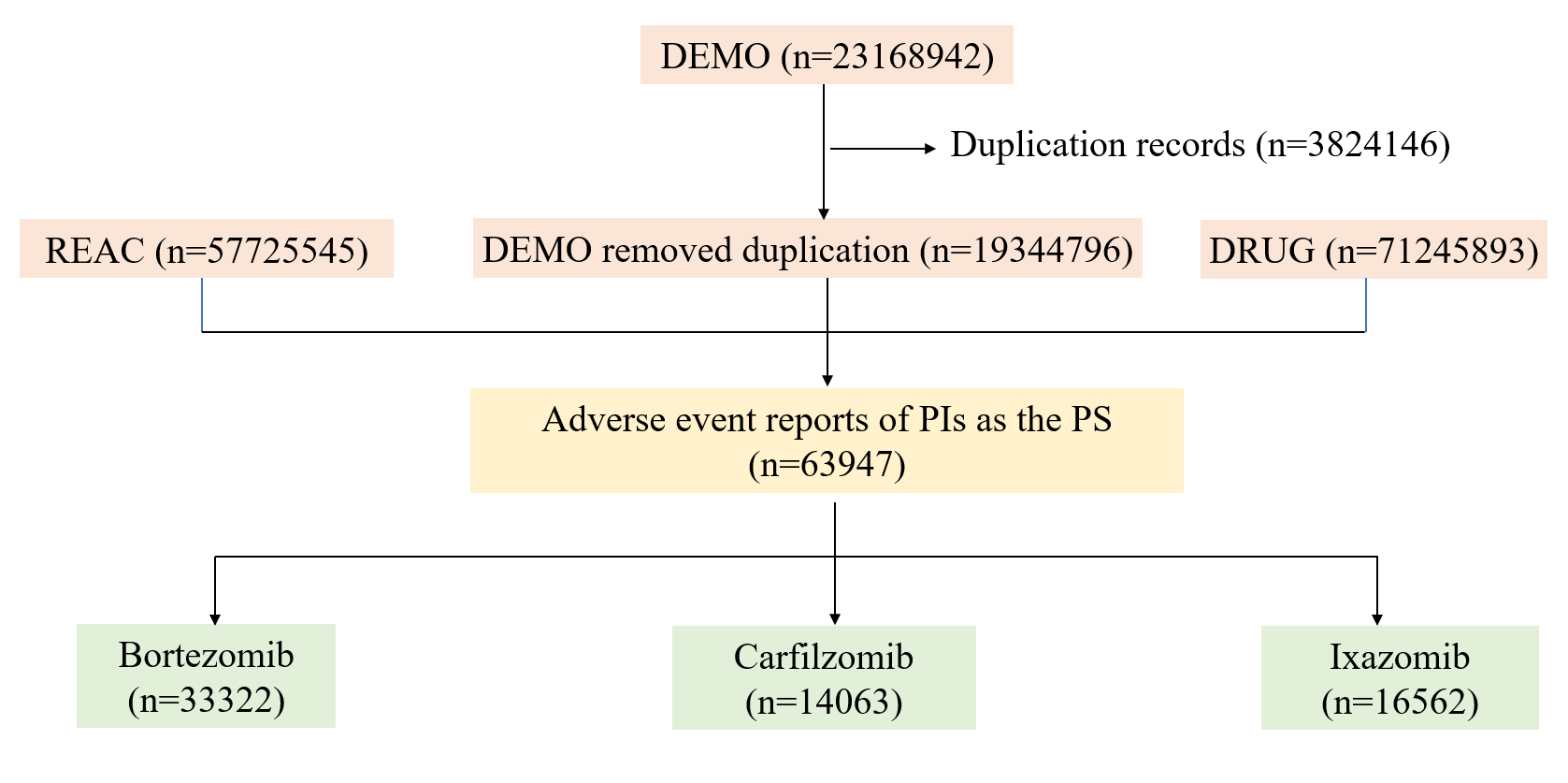
 **Supplementary Figure 1.** **Flowchart for screening in FAERS database**

(DEMO, REAC, and DRUG are subsets of data from the FAERS database; PI: proteasome inhibitors; PS: primary suspected)

**Supplementary Table 1. Four-grid table of ratio imbalance method**

|  | Target AEs | Other AEs | Total |
| --- | --- | --- | --- |
| Target drug | a | b | a+b |
| Other drugs | c | d | c+d |
| Total | a+c | b+d | a+b+c+d |

**Supplementary Table 2. Four major algorithms used for signal detection**

| Algorithms | Equation | Criteria |
| --- | --- | --- |
| ROR | ROR=ad/bc | lower limit of 95% CI>1, a≥3 |
|  | 95%CI=e^ln(ROR)±1.96(1/a+1/b+1/c+1/d)^0.5^ |  |
| PRR | PRR=a(c+d)/c/(a+b) | PRR≥2, χ^2^≥4, a≥3 |
|  | χ^2^=[(ad-bc)^2](a+b+c+d)/[(a+b)(c+d)(a+c)(b+d)] |  |
| BCPNN | IC=log_2_a(a+b+c+d)(a+c)(a+b) | IC025>0 |
|  | 95%CI= E(IC) ± 2V(IC)^0.5 |  |
| MGPS | EBGM=a(a+b+c+d)/(a+c)/(a+b) | EBGM05>2 |
|  | 95%CI=e^ln(EBGM)±1.96(1/a+1/b+1/c+1/d)^0.5^ |  |

Equation: a, number of reports containing both the target drug and target adverse drug reaction; b, number of reports containing other adverse drug reaction of the target drug; c, number of reports containing the target adverse drug reaction of other drugs; d, number of reports containing other drugs and other adverse drug reactions. 95%CI, 95% confidence interval; χ^2^, chi-squared; IC, information component; IC025, the lower limit of 95% CI of the IC; E(IC), the IC expectations; V(IC), the variance of IC; EBGM, empirical Bayesian geometric mean; EBGM05, the lower limit of 95% CI of EBGM.

**Supplementary Table 3 Top 10 neurological PTs with the highest** **frequency of occurrence**

| PT | Raw data | | | | | Data after removing thalidomide | | | | |
| --- | --- | --- | --- | --- | --- | --- | --- | --- | --- | --- |
|  | a | ROR (95%Cl) | PRR (χ^2^) | EBGM (EBGM05) | IC (IC025) | a | ROR (95%Cl) | PRR (χ^2^) | EBGM (EBGM05) | IC (IC025) |
| **Bortezomib** |  |  |  |  |  |  |  |  |  |  |
| Neuropathy peripheral | 2681 | 19.68 (18.93-20.47) | 19.16 (44791.45) | 18.60 (17.89) | 4.22 (4.15) | 2467 | 19.63 (18.85-20.45) | 19.11 (41204.77) | 18.6 (17.86) | 4.22 (4.15) |
| Polyneuropathy | 393 | 22.81 (20.62-25.23) | 22.72 (7866.62) | 21.93 (19.83) | 4.46 (4.23) | 329 | 20.62 (18.47-23.01) | 20.54 (5933.05) | 19.95 (17.88) | 4.32 (4.08) |
| Neurotoxicity | 221 | 8.72 (7.64-9.96) | 8.70 (1485.90) | 8.59 (7.52) | 3.10 (2.86) | 189 | 8.09 (7.01-9.34) | 8.07 (1157.16) | 7.99 (6.92) | 3.00 (2.73) |
| Peripheral sensory neuropathy | 171 | 19.95 (17.13-23.24) | 19.92 (2974.66) | 19.31 (16.58) | 4.27 (3.90) | 154 | 19.46 (16.58-22.85) | 19.43 (2614.94) | 18.90 (16.10) | 4.24 (3.85) |
| Neuralgia | 117 | 3.18 (2.65-3.81) | 3.18 (173.74) | 3.17 (2.64) | 1.66 (1.37) | 103 | 3.04 (2.51-3.69) | 3.04 (140.22) | 3.03 (2.50) | 1.60 (1.29) |
| Autonomic neuropathy | 96 | 70.16 (56.79-86.67) | 70.09 (5860.10) | 62.93 (50.94) | 5.98 (4.95) | 93 | 73.60 (59.4-91.20) | 73.52 (5984.72) | 66.24 (53.46) | 6.05 (4.98) |
| Posterior reversible encephalopathy syndrome | 87 | 5.65 (4.57-6.97) | 5.64 (329.21) | 5.60 (4.53) | 2.49 (2.10) | 80 | 5.64 (4.52-7.03) | 5.63 (302.33) | 5.59 (4.49) | 2.48 (2.08) |
| Guillain-barre syndrome | 72 | 10.15 (8.04-12.82) | 10.15 (583.91) | 10.00 (7.92) | 3.32 (2.81) | 64 | 9.79 (7.65-12.53) | 9.78 (497.32) | 9.65 (7.54) | 3.27 (2.73) |
| Peripheral motor neuropathy | 53 | 25.42 (19.31-33.47) | 25.41 (1192.75) | 24.43 (18.56) | 4.61 (3.69) | 47 | 24.38 (18.22-32.63) | 24.37 (1015.79) | 23.54 (17.59) | 4.56 (3.58) |
| Peripheral sensorimotor neuropathy | 45 | 26.16 (19.41-35.25) | 26.14 (1043.22) | 25.10 (18.63) | 4.65 (3.61) | 38 | 23.84 (17.25-32.96) | 23.83 (802.22) | 23.04 (16.66) | 4.53 (3.41) |
| **Carfilzomib** |  |  |  |  |  |  |  |  |  |  |
| Neuropathy peripheral | 248 | 4.12 (3.64-4.67) | 4.11 (581.79) | 4.10 (3.62) | 2.03 (1.83) | 238 | 4.07 (3.59-4.63) | 4.06 (547.32) | 4.05 (3.56) | 2.02 (1.81) |
| Polyneuropathy | 58 | 7.75 (5.99-10.04) | 7.74 (338.86) | 7.71 (5.95) | 2.95 (2.41) | 56 | 7.71 (5.93-10.02) | 7.70 (324.64) | 7.66 (5.89) | 2.94 (2.39) |
| Posterior reversible encephalopathy syndrome | 53 | 8.17 (6.24-10.70) | 8.16 (331.20) | 8.12 (6.20) | 3.02 (2.45) | 52 | 8.25 (6.28-10.84) | 8.24 (329.16) | 8.20 (6.24) | 3.04 (2.45) |
| Encephalopathy | 43 | 2.76 (2.04-3.72) | 2.75 (47.98) | 2.75 (2.04) | 1.46 (0.97) | 43 | 2.84 (2.10-3.83) | 2.84 (51.03) | 2.83 (2.10) | 1.50 (1.01) |
| Hypertensive encephalopathy | 7 | 18.09 (8.58-38.12) | 18.08 (111.58) | 17.87 (8.48) | 4.16 (1.50) | 6 | 15.93 (7.13-35.62) | 15.93 (83.07) | 15.77 (7.06) | 3.98 (1.24) |
| Intracranial mass | 5 | 6.03 (2.50-14.51) | 6.03 (20.88) | 6.01 (2.50) | 2.59 (0.53) | 5 | 6.21 (2.58-14.94) | 6.21 (21.74) | 6.18 (2.57) | 2.63 (0.55) |
| Autonomic neuropathy | 4 | 6.27 (2.35-16.73) | 6.27 (17.62) | 6.24 (2.34) | 2.64 (0.31) | 4 | 6.45 (2.42-17.23) | 6.45 (18.34) | 6.43 (2.41) | 2.68 (0.33) |
| Cauda equina syndrome | 4 | 7.97 (2.98-21.29) | 7.97 (24.24) | 7.93 (2.97) | 2.99 (0.44) | 3 | 6.14 (1.98-19.10) | 6.14 (12.87) | 6.12 (1.97) | 2.61 (0.02) |
| Central nervous system haemorrhage | 3 | 9.91 (3.19-30.86) | 9.91 (23.88) | 9.85 (3.17) | 3.30 (0.17) | 3 | 10.21 (3.28-31.78) | 10.21 (24.75) | 10.14 (3.26) | 3.34 (0.18) |
| Cerebral small vessel ischaemic disease | 3 | 7.50 (2.41-23.31) | 7.50 (16.80) | 7.46 (2.40) | 2.90 (0.06) | 3 | 7.72 (2.48-24.00) | 7.72 (17.45) | 7.68 (2.47) | 2.94 (0.08) |
| **Ixazomib** |  |  |  |  |  |  |  |  |  |  |
| Neuropathy peripheral | 675 | 9.68 (8.97-10.45) | 9.56 (5140.43) | 9.49 (8.80) | 3.25 (3.12) | 668 | 9.67 (8.95-10.44) | 9.54 (5076.87) | 9.48 (8.78) | 3.24 (3.11) |
| Paraesthesia | 252 | 3.10 (2.85-3.37) | 3.09 (143.42) | 3.09 (2.84) | 1.06 (0.87) | 249 | 3.09 (2.84-3.37) | 3.08 (140.44) | 3.08 (2.84) | 1.06 (0.87) |
| Dementia | 74 | 3.60 (2.87-4.53) | 3.60 (138.52) | 3.59 (2.86) | 1.84 (1.46) | 74 | 3.63 (2.89-4.57) | 3.63 (140.69) | 3.62 (2.88) | 1.86 (1.47) |
| Neurological symptom | 31 | 7.25 (5.09-10.32) | 7.24 (165.87) | 7.21 (5.06) | 2.85 (2.08) | 31 | 7.31 (5.14-10.41) | 7.31 (167.8) | 7.27 (5.11) | 2.86 (2.09) |
| Ataxia | 31 | 3.40 (2.39-4.83) | 3.39 (52.22) | 3.39 (2.38) | 1.76 (1.15) | 31 | 3.43 (2.41-4.87) | 3.42 (53.07) | 3.42 (2.40) | 1.77 (1.16) |
| Myoclonus | 30 | 3.30 (2.31-4.72) | 3.30 (47.90) | 3.29 (2.30) | 1.72 (1.10) | 30 | 3.33 (2.33-4.76) | 3.33 (48.70) | 3.32 (2.32) | 1.73 (1.11) |
| Peripheral sensory neuropathy | 17 | 3.90 (2.42-6.28) | 3.90 (36.56) | 3.89 (2.42) | 1.96 (1.06) | 17 | 3.94 (2.45-6.34) | 3.94 (37.11) | 3.93 (2.44) | 1.97 (1.07) |
| Chorea | 16 | 12.30 (7.52-20.14) | 12.30 (164.47) | 12.19 (7.45) | 3.61 (2.17) | 16 | 12.41 (7.59-20.31) | 12.41 (166.18) | 12.30 (7.51) | 3.62 (2.18) |
| Post herpetic neuralgia | 6 | 5.46 (2.45-12.18) | 5.46 (21.78) | 5.44 (2.44) | 2.44 (0.64) | 5 | 4.59 (1.91-11.05) | 4.59 (13.99) | 4.58 (2.90) | 2.19 (0.34) |
| Autonomic neuropathy | 5 | 6.67 (2.77-16.07) | 6.67 (23.99) | 6.64 (2.76) | 2.73 (0.59) | 4 | 5.38 (2.02-14.37) | 5.38 (14.20) | 5.36 (2.01) | 2.42 (0.22) |

**Supplementary Table 4 Top 10 neurological PTs with the highest signal strength**

| PT | Raw data | | | | | Data after removing thalidomide | | | | |
| --- | --- | --- | --- | --- | --- | --- | --- | --- | --- | --- |
|  | a | ROR(95%Cl) | PRR(χ^2^) | EBGM  (EBGM05) | IC(IC025) | a | ROR(95%Cl) | PRR(χ^2^) | EBGM  (EBGM05) | IC(IC025) |
| **Bortezomib** |  |  |  |  |  |  |  |  |  |  |
| Autonomic neuropathy | 96 | 70.16 (56.79-86.67) | 70.09 (5860.1) | 62.93 (50.94) | 5.98 (4.95) | 93 | 73.60 (59.40-91.20) | 73.52 (5984.72) | 66.24 (53.46) | 6.05 (4.98) |
| Hyponatraemic coma | 3 | 55.09 (16.90-179.63) | 55.09 (146.04) | 50.58 (15.51) | 5.66 (0.40) | 3 | 59.87 (18.36-195.21) | 59.87 (159.18) | 54.96 (16.86) | 5.78 (0.40) |
| Hyperintensity in brain deep nuclei | 5 | 43.29 (17.47-107.25) | 43.28 (192.76) | 40.46 (16.33) | 5.34 (1.19) | 5 | 47.04 (18.99-116.55) | 47.04 (210.28) | 43.97 (17.75) | 5.46 (1.20) |
| Neuralgic amyotrophy | 11 | 35.65 (19.41-65.49) | 35.65 (349.83) | 33.72 (18.36) | 5.08 (2.32) | 10 | 35.03 (18.54-66.18) | 35.03 (313.87) | 33.31 (17.63) | 5.06 (2.19) |
| Neuromyotonia | 3 | 31.34 (9.82-100.04) | 31.34 (83.79) | 29.85 (9.35) | 4.90 (0.37) | 3 | 34.06 (10.67-108.72) | 34.06 (91.54) | 32.44 (10.16) | 5.02 (0.38) |
| Phrenic nerve paralysis | 10 | 31.24 (16.54-58.98) | 31.24 (278.33) | 29.75 (15.76) | 4.89 (2.15) | 10 | 33.95 (17.98-64.10) | 33.94 (304.07) | 32.33 (17.12) | 5.01 (2.18) |
| Hypocalcaemic seizure | 3 | 30.81 (9.66-98.29) | 30.81 (82.35) | 29.37 (9.21) | 4.88 (0.37) | 3 | 33.49 (10.50-106.82) | 33.48 (89.97) | 31.91 (10.00) | 5.00 (0.38) |
| Peripheral sensorimotor neuropathy | 45 | 26.16 (19.41-35.25) | 26.14 (1043.22) | 25.10 (18.63) | 4.65 (3.61) | 38 | 23.84 (17.25-32.96) | 23.83 (802.22) | 23.04 (16.66) | 4.53 (3.41) |
| Peripheral motor neuropathy | 53 | 25.42 (19.31-33.47) | 25.41 (1192.75) | 24.43 (18.56) | 4.61 (3.69) | 47 | 24.38 (18.22-32.63) | 24.37 (1015.79) | 23.54 (17.59) | 4.56 (3.58) |
| Polyneuropathy | 393 | 22.81 (20.62-25.23) | 22.72 (7866.62) | 21.93 (19.83) | 4.46 (4.23) | 329 | 20.62 (18.47-23.01) | 20.54 (5933.05) | 19.95 (17.88) | 4.32 (4.08) |
| **Carfilzomib** |  |  |  |  |  |  |  |  |  |  |
| Hypertensive encephalopathy | 7 | 18.09 (8.58-38.12) | 18.08 (111.58) | 17.87 (8.48) | 4.16 (1.50) | 6 | 15.93 (7.13-35.62) | 15.93 (83.07) | 15.77 (7.06) | 3.98 (1.24) |
| Central nervous system haemorrhage | 3 | 9.91 (3.19-30.86) | 9.91 (23.88) | 9.85 (3.17) | 3.30 (0.17) | 3 | 10.21 (3.28-31.78) | 10.21 (24.75) | 10.14 (3.26) | 3.34 (0.18) |
| Posterior reversible encephalopathy syndrome | 53 | 8.17 (6.24-10.70) | 8.16 (331.20) | 8.12 (6.20) | 3.02 (2.45) | 52 | 8.25 (6.28-10.84) | 8.24 (329.16) | 8.20 (6.24) | 3.04 (2.45) |
| Cauda equina syndrome | 4 | 7.97 (2.98-21.29) | 7.97 (24.24) | 7.93 (2.97) | 2.99 (0.44) | 3 | 6.14 (1.98-19.10) | 6.14 (12.87) | 6.12 (1.97) | 2.61 (0.02) |
| Polyneuropathy | 58 | 7.75 (5.99-10.04) | 7.74 (338.86) | 7.71 (5.95) | 2.95 (2.41) | 56 | 7.71 (5.93-10.02) | 7.70 (324.64) | 7.66 (5.89) | 2.94 (2.39) |
| Cerebral small vessel ischaemic disease | 3 | 7.50 (2.41-23.31) | 7.50 (16.80) | 7.46 (2.40) | 2.90 (0.06) | 3 | 7.72 (2.48-24.00) | 7.72 (17.45) | 7.68 (2.47) | 2.94 (0.08) |
| Autonomic neuropathy | 4 | 6.27 (2.35-16.73) | 6.27 (17.62) | 6.24 (2.34) | 2.64 (0.31) | 4 | 6.45 (2.42-17.23) | 6.45 (18.34) | 6.43 (2.41) | 2.68 (0.33) |
| Intracranial mass | 5 | 6.03 (2.50-14.51) | 6.03 (20.88) | 6.01 (2.50) | 2.59 (0.53) | 5 | 6.21 (2.58-14.94) | 6.21 (21.74) | 6.18 (2.57) | 2.63 (0.55) |
| Neuropathy peripheral | 248 | 4.12 (3.64-4.67) | 4.11 (581.79) | 4.10 (3.62) | 2.03 (1.83) | 238 | 4.07 (3.59-4.63) | 4.06 (547.32) | 4.05 (3.56) | 2.02 (1.81) |
| Peripheral motor neuropathy | 3 | 3.30 (1.06-10.24) | 3.30 (4.79) | 3.29 (2.06) | 1.72 (0.38) | 3 | 3.39 (1.09-10.54) | 3.39 (5.06) | 3.39 (2.09) | 1.76 (0.36) |
| **Ixazomib** |  |  |  |  |  |  |  |  |  |  |
| Burning feet syndrome | 3 | 16.61 (5.32-51.91) | 16.61 (43.43) | 16.40 (5.25) | 4.06 (2.60) | 3 | 16.76 (5.36-52.37) | 16.76 (43.86) | 16.55 (5.30) | 4.05 (0.30) |
| Chorea | 16 | 12.30 (7.52-20.14) | 12.3 (164.47) | 12.19 (7.45) | 3.95 (3.24) | 16 | 12.41 (7.59-20.31) | 12.41 (166.18) | 12.30 (7.51) | 3.62 (2.18) |
| Neuropathy peripheral | 675 | 9.68 (8.97-10.45) | 9.56 (5140.43) | 9.49 (8.80) | 3.15 (3.03) | 668 | 9.67 (8.95-10.44) | 9.54 (5076.87) | 9.48 (8.78) | 3.24 (3.11) |
| Neurological symptom | 31 | 7.25 (5.09-10.32) | 7.24 (165.87) | 7.21 (5.06) | 3.05 (2.54) | 31 | 7.31 (5.14-10.41) | 7.31 (167.80) | 7.27 (5.11) | 2.86 (2.09) |
| Autonomic neuropathy | 5 | 6.67 (2.77-16.07) | 6.67 (23.99) | 6.64 (2.76) | 2.86 (1.67) | 4 | 5.38 (2.02-14.37) | 5.38 (14.20) | 5.36 (2.01) | 2.42 (0.22) |
| Senile dementia | 3 | 5.94 (1.91-18.47) | 5.94 (12.26) | 5.91 (2.90) | 2.74 (1.29) | 3 | 5.99 (1.93-18.63) | 5.99 (12.41) | 5.97 (2.92) | 2.58 (0.04) |
| Post herpetic neuralgia | 6 | 5.46 (2.45-12.18) | 5.46 (21.78) | 5.44 (2.44) | 2.54 (1.44) | 5 | 4.59 (1.91-11.05) | 4.59 (13.99) | 4.58 (2.90) | 2.19 (0.34) |
| Peripheral sensorimotor neuropathy | 4 | 4.54 (1.70-12.11) | 4.54 (10.99) | 4.52 (2.69) | 2.18 (0.11) | 3 | 3.43 (1.10-10.65) | 3.43 (5.15) | 3.42 (2.10) | 1.77 (0.35) |
| Peripheral sensory neuropathy | 17 | 3.90 (2.42-6.28) | 3.90 (36.56) | 3.89 (2.42) | 1.96 (1.06) | 17 | 3.94 (2.45-6.34) | 3.94 (37.11) | 3.93 (2.44) | 1.97 (1.07) |
| Dementia | 74 | 3.60 (2.87-4.53) | 3.60 (138.52) | 3.59 (2.86) | 1.84 (1.46) | 74 | 3.63 (2.89-4.57) | 3.63 (140.69) | 3.62 (2.88) | 1.86 (1.47) |
